# Calcineurin-Modulated Antimicrobial Peptide Expression Is Required for the Development of *Helicoverpa armigera*

**DOI:** 10.3389/fphys.2019.01312

**Published:** 2019-10-17

**Authors:** Jizhen Wei, Linhong Li, Shuangyan Yao, Shuo Yang, Shuai Zhou, Xiaoguang Liu, Mengfang Du, Shiheng An

**Affiliations:** State Key Laboratory of Wheat and Maize Crop Science, College of Plant Protection, Henan Agricultural University, Zhengzhou, China

**Keywords:** calcineurin, AMP, IMD pathway, relish, *Helicoverpa armigera*

## Abstract

*Helicoverpa armigera* is a universal pest around the world that has been extensively used as a model organism for agricultural pests. Calcineurin (CAN) is an important Ca^2+^-dependent phosphatase that is participated in various biological pathways. Here, we revealed that CAN inhibition significantly arrested *H. armigera* larval development by reducing larvae weight, prolonging development time and reducing pupate rates. Furthermore, CAN serves as an immune activator and regulates antimicrobial peptide (AMP; cecropin D, attacin, and gloverin) expression by binding with relish transcript factor in *H. armigera*. This study provides a potential target to control *H. armigera* by using synergistic agents for pesticides or plant-mediated RNA interference technology.

## Introduction

Calcineurin (CAN) belongs to the protein phosphatase 2B family and acts as a Ca^2+^-dependent phosphatase. CAN is ubiquitously expressed in most mammalian tissues, and exerts different functions in different tissues (Furman and Norris, 2014). Studies have reported that CAN is predominantly expressed in the brain and plays important roles in various neuronal functions and disorders. This is due to CAN’s vital functions in Ca^2+^ homeostasis, synaptic plasticity, receptor signaling, and transcription regulation (Jackson et al., 2018). Furthermore, CAN is also found to be expressed in lymphocytes and macrophages, in which CAN has been confirmed to be took part in innate immunity (Bueno et al., 2002; Kang et al., 2007). In addition, In human endothelial cells, CAN functions as a mediator of anaphylaxis (Ballesteros-Martinez et al., 2017). These studies provide key evidence that CAN is a vital signaling molecule that performs a variety of functions in different biological pathways (Liu et al., 1991; Jackson et al., 2018).

In insects, studies on the functions of CAN have mostly been performed in the model organism *Drosophila melanogaster*. In *Drosophila*, CAN plays a crucial role in myofilament formation and troponin I isoform transition which affects the development of flight muscles (Gajewski et al., 2006). Interestingly, CAN in *Drosophila* has also been demonstrated to participate in circadian rhythms regulation (Kweon et al., 2018), axonal transport (Shaw et al., 2013), mitochondrial function (Chang and Min, 2005), olfactory associative learning and long-term memory (Chang et al., 2003), courtship behaviors (Ejima et al., 2004; Sakai and Aigaki, 2010), and female reproductive activities (Ejima et al., 2004; Horner et al., 2006; Takeo et al., 2010). Besides, *Drosophila* CAN has also been identified to play a role in immune response. When *Drosophila* were infected with gram-negative bacteria, CAN activity increased (Li and Dijkers, 2015). This promoted the product of relish, a key transcription factor of the immune deficiency (IMD) pathway in the innate immunity, which finally induces antimicrobial gene expression (Dijkers and O’Farrell, 2007; Li and Dijkers, 2015).

Besides *Drosophila*, relatively few studies have focused on CAN role in other insect species. In the Lepidoptera adult, *Bombyx mori*, CAN is localized in the cytoplasm of pheromone-producing cells and regulates the production of sex pheromone (Yoshiga et al., 2002). In *Helicoverpa armigera*, CAN in adults mediates female attraction to males (Zhao et al., 2018). Furthermore, CAN has been confirmed to activate acetyl CoA carboxylase through dephosphorylation, and thus regulate sex pheromone biosynthesis and female mating acceptance in *H. armigera* (Du et al., 2017; Zhao et al., 2018). While these studies deepen our understanding of CAN function in adults, CAN function in larvae remains elusive and lacking.

*Helicoverpa armigera* is a universal pest around the world, and many measures have been taken to control this pest, including planting transgenic crops expressing a chemical pesticide, *Bacillus thuringiensis* (BT) (Downes et al., 2017; Wei et al., 2018). Although these measures work to some extent, they also present many problems, such as insect resistance. In order to find better methods to control *H. armigera*, it is critical to study the associated genes that involved in certain physiological process, such as genes involved in the immune process. Insect and mammalian immune systems share many similarities (Kaneko et al., 2004; Kaneko and Silverman, 2005). Insects depend solely upon the innate immune responses to survive. In the IMD pathway, the expression of antimicrobial genes is required to respond to gram-negative bacteria, thus making CAN an attractive target to control *H. armigera* CANs from different species share high amino acid identity, indicating they may share similar functions (Chen and Zhang, 2013; Zhao et al., 2018). Whether the IMD pathway exists in *H. armigera* larvae has never been addressed.

In this study, CAN from *H. armigera* (*HaCAN*) was found to be widely distributed in different developmental stages and different tissues, especially in fourth and fifth instars. Feeding of *H. armigera* larvae with FK506 significantly affected their development. FK506 treatments also significantly attenuated the expression of antimicrobial peptide (AMP) transcripts (cecropin D, attacin, and gloverin) caused by infection with gram-negative bacteria. For the first time, *HaCAN* was confirmed to be involved in larval development. *HaCAN* was also found to interact with relish. The present study reveals a potential target for synergistic agents such as pesticides or plant-mediated RNA interference (RNAi) technology, to control *H. armigera*.

## Materials and Methods

### Insect Rearing

Larvae of *H. armigera* were purchased from the Henan Jiyuan Baiyun Industry Co., Ltd. Larvae were reared on artificial diet at 26 ± 1°C, 60 ± 10% relative humidity (RH), and a photoperiod of 16L:8D h (Du et al., 2017).

### Tissue Sampling

Larvae on the second day of every instar (from first to fifth) were collected with three replicates. The collected samples were used for subsequent RNA extraction to analyze the developmental expression profile of *HaCAN* (GenBank: KR185962.1).

In order to investigate distributions of *HaCAN* in the different tissues (including midgut, malpighian tubes, salivary glands, fat body, hemocyte, central nervous, and epidermis), the tissues were harvested from more than five larvae on the ice. This served as one biological replicate.

Expression patterns of the endogenous AMPs [cecropin D (GenBank: EU041763.1), attacin (GenBank: AY948540.1), and gloverin (GenBank: KT346373.1)] in the midgut, fat body and epidermis were analyzed by quantitative real-time PCR after tacrolimus (FK506) treatment. Third instar larvae were fed with different doses of FK506 (50, 100, and 200 μM). DMSO (FK506 solvent) treatment was used as control. Midgut, fat body, and epidermis were dissected from 10 larvae and served as one biological replicate. The expression levels of AMPs in larvae were tested 72 h after FK506 (or DMSO) treatments.

Expressions of endogenous AMPs (cecropin D, attacin, and gloverin) in midgut, fat body, and epidermis were analyzed by quantitative real-time PCR after FK506 treatment or *Escherichia coli* injection. The FK506 inhibitor was dissolved in DMSO. Either 50 μM FK506 or DMSO was fed to third instar larvae. 72 h after FK506 (or DMSO) treatments larvae were then injected with either 3 μL *E. coli* cells suspended in PBS (4 × 10^5^ cell/μL) or 3 μL PBS. The larvae were incubated for either 1 h or 3 h, after which, the midgut, fat body and epidermis was harvested. All the collected samples were quickly frozen in liquid nitrogen and stored at −80°C for subsequent RNA extraction. Ten larvae were used for one biological replicate.

All the above treatments were carried out with three biological replicates.

### Quantitative Real-Time PCR Analysis of *HaCAN* Expression

Total RNA was extracted from each sample prepared above with TRIzol reagent (Invitrogen, Carlsbad, CA, United States) according to the manufacturer’s instructions. All RNA samples were analyzed by UV spectrophotometry (NanoDrop; Thermo Scientific). A260/A280 ratio of all RNA samples were >1.8. All RNA samples were run on 1% agarose gel to ensure the quality of RNA (data not shown). After that, 1 μg of each DNase I-treated RNA sample was reversely transcribed into first strand cDNA using the PrimeScript RT reagent kit with gDNA Eraser (TaKaRa, Daliang, China). The above first-strand cDNA was then used as the template for qRT-PCR. The primers used for qRT-PCR analysis are listed in Table 1. Amplification efficiency of each primer pair was examined on an Applied Biosystems 7500 Fast Real-Time PCR system and calculated using the formula, PCR efficiency = 10^–1/slope^ − 1 (Bustin et al., 2009). All the primer pairs had an amplification efficiency between 97 and 98% (Table 1). QRT-PCR analysis of *HaCAN* and *18S* (internal reference gene; Zhang S. et al., 2015) were performed individually in a 12 μL reaction system containing 6 μL 2 × SYBR^®^ Premix Ex TaqTM II (Eurogentec, Fremont, CA, United States), 5 μM gene-specific forward primer and reverse primer (0.5 μL each), 1 μL template cDNA, and 4 μL nuclease-free water using an Applied Biosystems 7500 Fast Real-Time PCR system. Thermocycler conditions consisted of an initial denaturation for 3 min at 95°C, followed by 35 cycles of denaturation at 95°C for 15 s and annealing/extension at 58°C for 30 s. A melting curve was generated after the termination of PCR cycles to ensure free of junk products. For each gene in each treatment, at least three biological replicates were tested with three technical replicates. The mRNA expression was quantified using the comparative cross threshold method (CT, the PCR cycle number that crosses the signal threshold) (Livak and Schmittgen, 2001). The CT result of the 18S gene was subtracted from the CT result of the target gene to obtain a ΔCT. Normalized fold changes in target gene mRNA expression were expressed as 2^–ΔΔCT^, where ΔΔCT is equal to ΔCT treated sample – substracted from the ΔCT control (Schmittgen and Livak, 2008).

**TABLE 1:** Primers used in this study.

**Gene**	**Primer sequences (5′ → 3′)**	**Sizes of amplicons**	**Amplification efficiency (%)**	**Annealing temperature (°C)**
**Real-time PCR**				
18S-RT	F: GCATCTTTCAAATGTCTGC	230 bp	97	58
	R: TACTCATTCCGATTACGAG			
CNA-RT	F: AAGATGCTGGTTACCGAATGT	211 bp	99	58
	R: AAGGGACCAGGTAAAGACAT			
	R: ACTTTGAACATTATTGCTTA			
Cecropin D-RT	F: TGTTTGCTTGGTTCTGGTT	156 bp	99	58
	R: TTTTCTTCCGAGCTGTCGT			
Attacin-RT	F: CTGCCAATAACTGGTGACGAT	201 bp	98	58
	R: CATCGTGGAACAGATTCGCAT			
Gloverin-RT	F: TTAGCCCTTACGGTGACAG	178 bp	98	58
	R: TCCAAGTTGCCTCCCGCTGAT			
**Vector construction**				
pDHB1-relish	F: TGTCTCCTAAGAACGCGGCCATTACGGCCATGTCTACAAGCAGTGACC	2835 bp	NA	60
	R: GGGATCCCCCCCGACATGGCCGAGGCGGCATCGATATACCTCCGTATGA			
pPR3-N-CNA	F: TGTTCCAGATTACGCTGGATCCATGTCCGGGAGCAATGAT	1488 bp	NA	60
	R: ACTAATTACATGACTCGAGGTCGACTCACGAATGTGCGTTGCT			

The expression levels of cecropin D, attacin, and gloverin in *H. armigera* larvae were analyzed using qRT-PCR method as the above mentioned after treatments with FK506, *E. coli*, FK506 + *E. coli*. The primers of cecropin D, attacin, and gloverin are listed in the Table 1.

### Effects on *H. armigera* Development After Feeding With FK506

Tacrolimus (FK506) is a special CAN inhibitor, which inhibits CAN phosphatase activity and affects the insect immunity (Liu et al., 1991, 1992; Dijkers and O’Farrell, 2007). Three concentrations of FK506 (50, 100, and 200 μM) were used to evaluate its effect on the development of *H. armigera* larvae (DMSO, FK506 solvent, was set as a control). Third instar larvae, 12 h before molting, were transferred onto the artificial diet that was mixed with either FK506 or DMSO. For each treatment, five replicates (30 third instar larvae per replicates × 5 replicates = 150 larvae total per concentration for each treatment) were employed.

In these experiments, the weights of the treated insects were recorded every other day. After 7 days, dead insects were counted to calculate the mortalities. The days, when the treated larvae began to pupate, were record as the larvae’s development time. Successfully pupated insects were counted to calculate pupation rates. For the healthy pupae, the numbers of successfully emerged adults were counted to calculate the adult emergence rate.

### Protein–Protein Interactions Between *HaCAN* and *Harelish*

A full open reading frame of *Harelish* (GenBank: KR185962.1) and *HaCAN* were PCR-amplified using the gene-specific primers pDHB1-N-*Harelish* (F and R) and pPR3-N-CAN (F and R) from relish-pGEMT and CAN-pGEMT (Table 1) (Du et al., 2017), respectively. The PCR product of *Harelish* was cloned into bait vector pDHB1-N (Dualsystems Biotech), and *HaCAN* was cloned into pPR3-N vector (Dualsystems Biotech).

Several colonies of NMY51 (Dualsystems Biotech) were picked from YPAD plates and incubated in 3 mL YPAD media at 30°C, while shaking at 250 rpm for 8–12 h. Upon the culture reaching an OD600 of 0.15–0.3 (about 16–20 h), the culture was centrifuged at 700 × *g* for 5 min, the supernatant was discarded and pellet was resuspended in 100 mL YPAD. The bacterium solution was centrifuged at 700 × *g* for 5 min and washed again using 30 mL ddH_2_O. The collected bacteria were then resuspended in 600 μL 1.1 × TE/LiAc solution [1.1 × TE/LiAc (10 mL): 1.1 mL 1 M LiAc, 1.1 mL 10 × TE Buffer, 8.8 mL ddH_2_O].

A 5 μL carrier DNA (10 μg/mL) were boiled for 5 min and added into a 1.5 mL eppendorf tube. 500 μL PEG/LiAc solution [1 × PEG/LiAc (10 mL): 8 mL 50% PEG3350, 1 mL 10 × TE Buffer, 1 mL 1 M LiAc] was added with 50 μL NMY51 cells to the carrier DNA tube then mixed with the corresponding plasmids. Four treatments were prepared including 100 ng pDHB1-largeT and 100 ng pDSL-p53, 100 ng pDHB1-relish and 100 ng pOst1-NubI, 100 ng pDHB1-relish and 100 ng pPR3-N, and 100 ng pDHB1-relish and 100 ng pPR3-N-CAN. Each treatment was incubated at 30°C with shaking every 10 min. 20 μL DMSO was added to each tube and the tubes were transferred to a 42°C water bath and incubated for 15 min while, vortexing every 5 min. The solutions were then centrifuged at 700 × *g* for 5 min and the cell pellets were dissolved in 1 mL 0.9% NaCl. 100 μL suspension was taken from each transformation and plated onto SD-Leucine-Tryptophan plate (DDO; Clontech), and SD-Leucine-Tryptophan-Histidine-Adenine plates (QDO; Clontech). All plates were incubated for 5 days at 30°C (Yu et al., 2015).

Five days after the transformation of cDNA library, nine white colonies from the QDO plates were transferred into 1.5 mL eppendorf tubes containing 500 μL 0.9% NaCl. 0.9% NaCl was used to normalize the OD600 of each treatment to the same value. 1 μL of transformed solution was added onto SD/-LT/X (SD-Leucine-Tryptophan + 40 mg/L X-α-Gal media) (X-α-Gal soluted in N, N-dimethylformamide), SD/-LTH/X (SD-Leucine-Tryptophan-Histidine + 40 mg/L X-α-Gal media), SD/-LTHA/X (SD-Leucine-Tryptophan-Histidine-Adenine + 40 mg/L X-α-Gal media), SD/-LTHA/X/10 mM3-AT (SD-Leucine-Tryptophan-Histidine-Adenine + 40 mg/L X-α-Gal media + 3 mM 3-AT) and SD/-LTHA/X/10 mM 3-AT (SD-Leucine-Tryptophan-Histidine-Adenine + 40 mg/L X-α-Gal media + 10 mM 3-AT) plates. These plates were used to determine the threshold of selection which was increased by either removal of essential amino acids or by using 3-AT, a competitive inhibitor of the *his* gene product and the highest selection stress used in these four plates (Fetchko and Stagljar, 2004).

### Statistical Analysis

Significant differences in the relative expression levels of *HaCAN* in different instars and different tissues were compared using Tukey’s test with *P* < 0.05 [analysis of variance (ANOVA) and DPS7.05]. Significant differences in the relative expression levels of cecropin D, attacin, and gloverin in different treatments were compared using Tukey’s test with *P* < 0.05 (ANOVA and DPS7.05).

## Results

### Developmental Expression Pattern of *HaCAN* in Larvae

**FIGURE 1:**
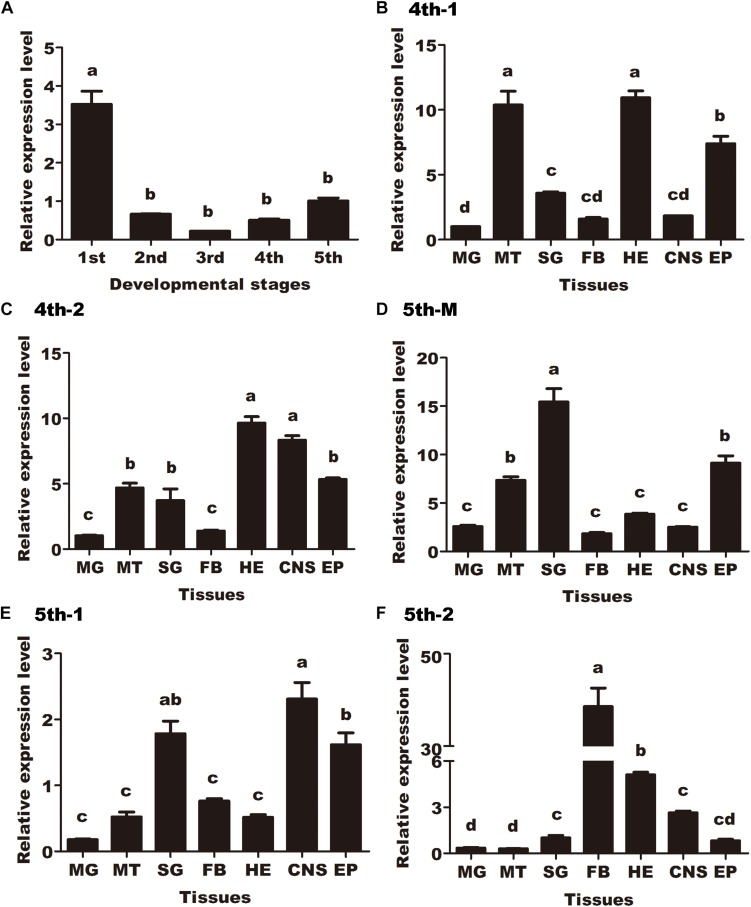
Developmental expression pattern and spatial distributions of *Helicoverpa armigera calcineurin A* (*HaCNA*) in larvae as determined by quantitative real-time PCR. **(A)** Relative expression levels of *HaCNA* in first-instar larvae (1st), second-instar larvae (2nd), third-instar larvae (3rd), fourth-instar larvae (4th), and fifth-instar larvae (5th). **(B)** Relative expression levels of *HaCNA* in different tissues (MG, midgut; MT, malpighian tubes; SG, salivary glands; FB, fat body; HE, hemocyte; CNS, central nervous; EP, epidermis) in the first day of 4th larvae. **(C)** Relative expression levels of *HaCNA* in different tissues in the second day of 4th larvae. **(D)** Relative expression levels of *HaCNA* in different tissues in fifth instar molting stage. **(E)** Relative expression levels of *HaCNA* in different tissues in the first day of 5th larvae. **(F)** Relative expression levels of *HaCNA* in different tissues in the second day of 5th larvae. Statistically significant differences for experimental comparisons are indicated by different small letters [analysis of variance (ANOVA) and Tukey’s test, *P* < 0.05; DPS7.05]. Values shown are means and standard errors.

To investigate the developmental expression pattern of *HaCAN* in different instars (first-, second-, third-, fourth-, and fifth-instar larvae), qRT-PCR was employed and results revealed that *HaCAN* was ubiquitously expressed in all of the examined stages. The expression level of *HaCAN* reached its peak at the first-instar larvae, followed by fifth-instar larvae (Figure 1A).

The spatial distributions of *HaCAN* were investigated in different tissues (midgut, malpighian tubes, salivary glands, fat body, hemocytes, central nervous system, and epidermis) during different developmental stages (the first and second day of fourth instar larvae, molting day, and the first and second day of fifth-instar larvae). The distribution patterns of *HaCAN* in different tissues varied (Figures 1B–F), which generally exhibited higher expression levels in the epidermis during the first and second day in fourth instar larvae, the molting day and the first day in fifth instar larvae. Interestingly, *HaCAN* transcriptional level showed a sudden increase on the second day in fifth instar larvae (Figures 1B–F).

### Effect of CAN Inhibitor on *H. armigera* Development

**FIGURE 2:**
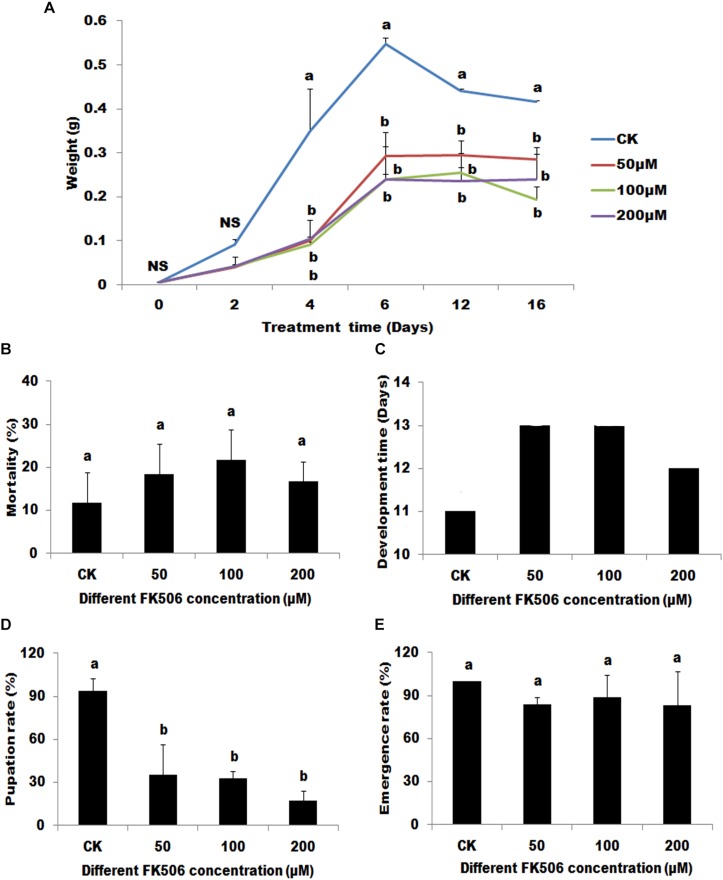
Effects of the calcineurin (CN) inhibitor (FK506) on the *Helicoverpa armigera*. The larvae were treated begin at the first day of 3rd. **(A)** The effect of FK506 on the larvae weight. Larvae were fed with different FK506 concentrations (50, 100, and 200 μM) (DMSO as buffer control). **(B)** The mortality of larvae 7 days after different treatments. **(C)** The developmental time of the different treatments from the first day of 3rd instar larvae to began to pupate. **(D)** The pupation rate of the different treatments. **(E)** The emergence rate of the successfully pupate insects under the different treatments. Statistically significant differences for experimental comparisons are indicated by different small letters [analysis of variance (ANOVA) and Tukey’s test, *P* < 0.05; DPS7.05]. Values shown are means and standard errors.

Four days of FK506 treatment caused significant decreases in body weight compared with controls (DMSO treatment). However, no significant differences were observed between the different FK506 concentrations used (Figure 2A).

Although seven days of FK506 treatments did not lead to significant changes in larvae mortalities, FK506 treatments significantly arrested the development of the larvae (Figures 2B,C). Pupation was delayed by 1-2 days and the pupation rates were significantly decreased (35.00, 32.47, and 17.36% after treatments with 50, 100, and 200 μM FK506, respectively, versus 93.75% after DMSO treatment) (Figures 2C,D). However, FK506 treatments exhibited no effect on the emergence rate of pupae (Figure 2E).

### Protein–Protein Interactions Between *HaCAN* and *Harelish*

**FIGURE 3:**
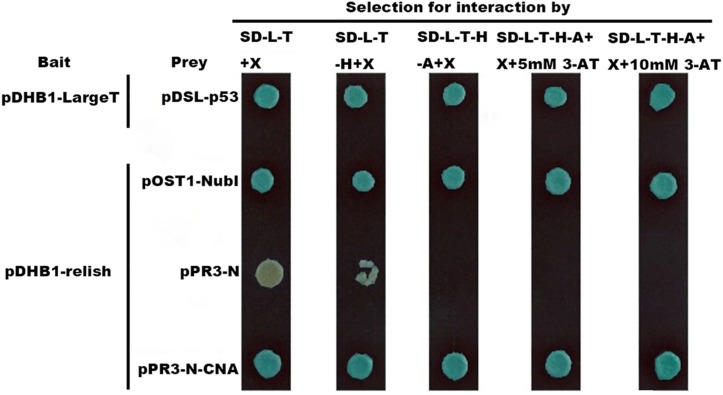
The interactions between *HaCNA* and *Harelish* based on yeast two-hybrid system. pDHB1 vector serves as the bait which is fused with the C-terminal half of ubiquitin (Cub) and an artificial transcription factor (LexA-VP6), while in the vector of pPR3-N are fused with the N-terminal half of ubiquitin (NubG, replacement of Ile-13 of wildtype NubI by glycine decreases the affinity between Nub and Cub.). The treatment of pDHB1-LargeT & pDSL-p53 was positive control to show this yeast two-hybrid system works well. SD-L-T + X: SD-Leucine-Tryptophan + 40 mg/L X-α-Gal media (X-α-Gal soluted in N,N-dimethylformamide); SD-L-T-H + X: SD-Leucine-Tryptophan-Histidine + 40 mg/L X-α-Gal media; SD-L-T-H-A + X: SD-Leucine-Tryptophan-Histidine-Adenine + 40 mg/L X-α-Gal media; SD-L-T-H-A + X + 5 mM 3-AT: SD-Leucine-Tryptophan-Histidine-Adenine + 40 mg/L X-α-Gal media + 5 mM 3-AT; SD-L-T-H-A + X + 10 mM 3-AT: SD-Leucine-Tryptophan-Histidine-Adenine + 40 mg/L X-α-Gal media + 10 mM 3-AT.

To test yeast two-hybrid system, pDHB1-LargeT and pDSL-p53 were transformed as a positive control and were able to see growth on the selection plates, which showed that the yeast two-hybrid system works well (Figure 3). The transformation of pDHB1-N-relish and pOst1-NubI grew well on SD-Leucine-Tryptophan (DDO; Clontech) and SD-Leucine-Tryptophan-Histidine-Adenine (QDO; Clontech) plates, respectively. Moreover, the transformation of pBT3-N-relish & pOst1-NubI turned blue in the β-galactosidase assay on SD/-LT/X, SD/-LTH/X, SD/-LTHA/X, SD/-LTHA/X/3mM 3-AT and SD/-LTHA/X/10 mM 3-AT plates, respectively (Figure 3). These experiments demonstrated that pBT3-N-relish plasmids worked well. The vector pPR3-N expresses the N-terminal half of ubiquitin (NubG) and transformation of pBT3-N-relish and pPR3-N displayed less growth on normal selection plate while there were no clonies on QDO plates. There were also no blue colonies present in the β-galactosidase assay on SD/-LT/X, SD/-LTH/X, SD/-LTHA/X, SD/-LTHA/X/10 mM 3-AT plates (Figures 1, 2). These results showed that the clones of pDHB1-N-relish and pPR3-N on QDO plates were false positives, and that the split-ubiquitin membrane yeast two-hybrid system can be used to identify interactions between *HaCAN* and *Harelish*. The transformation of pDHB1-N-relish & pPR3-N-CAN grew well on both DDO plates and QDO plates. Moreover, the colonies resulting from this transformation turned blue in a β-galactosidase assay on SD/-LT/X, SD/-LTH/X, SD/-LTHA/X, SD/-LTHA/X/3 mM 3-AT and SD/-LTHA/X/10 mM 3-AT plates, indicating that *HaCAN* and *Harelish* interact directly (Figure 3).

### *HaCAN* Regulates AMPs Expression

**FIGURE 4:**
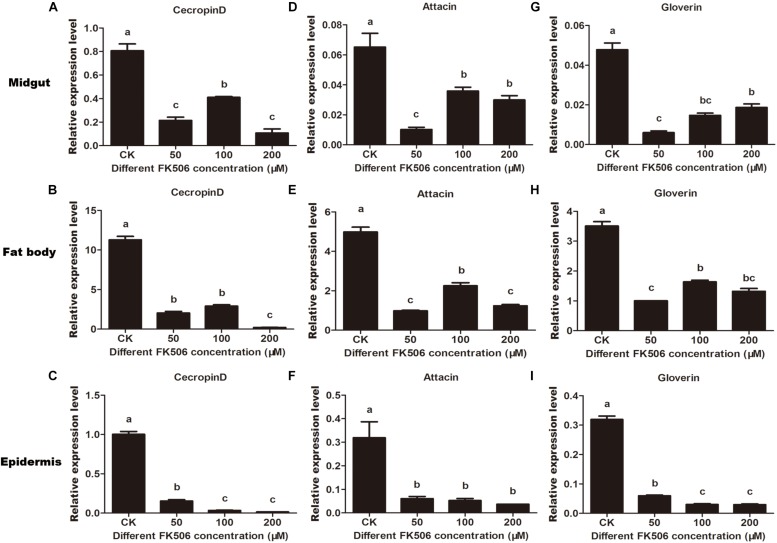
Expression levels of endogenous antimicrobial peptides (cecropin D, attacin, and gloverin) in midgut, fat body and epidermis after feeding FK506 (0, 50, 100, and 200 μM) were analyzed by quantitative real-time PCR. **(A–C)** Represent the relative expression levels of cecropin D in the migut, fat body, and epidermis, respectively. **(D–F)** Represent the relative expression levels of attacin in the migut, fat body and epidermis, respectively. **(G–I)** Represent the relative expression levels of gloverin in the migut, fat body, and epidermis, respectively. Values shown are means and standard errors. Statistically significant differences for experimental comparisons are indicated by different small letters [analysis of variance (ANOVA) and Tukey’s test, *P* < 0.05; DPS7.05].

To investigate the regulation of AMPs expression by *HaCAN*, the CAN inhibitor FK506 was used. The results demonstrated that HaCAN inhibition with different FK506 resulted in a significant decrease in the expression levels of several AMPs, including cecropin D, attacin, and gloverin in the midgut, fat body and epidermis of larvae (Figure 4).

**FIGURE 5:**
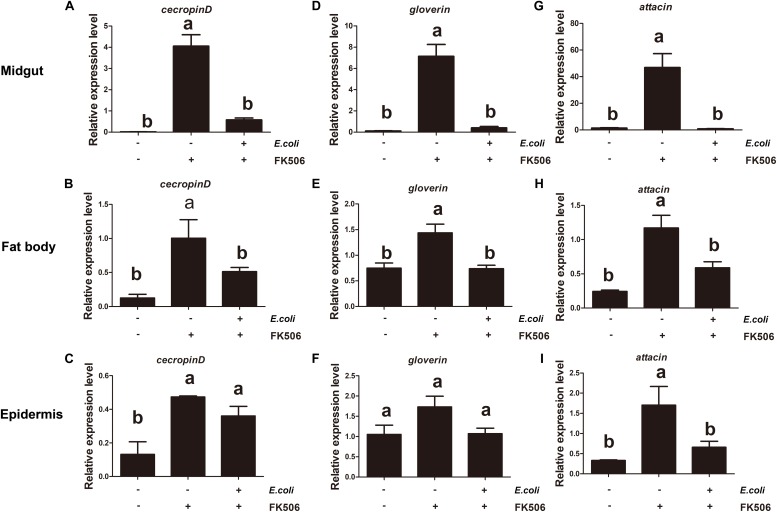
Expression levels of endogenous antimicrobial peptides (cecropin D, attacin, and gloverin) in the midgut, fat body, and epidermis in response to *Escherichia coli* infection and *E. coli* + FK506 in fourth instar *H. armigera* after 1 h treatment. **(A–C)** Represent the relative expression levels of cecropin D in the migut, fat body, and epidermis, respectively. **(D–F)** Represent the relative expression levels of attacin in the migut, fat body, and epidermis, respectively. **(G–I)** Represent the relative expression levels of gloverin in the migut, fat body, and epidermis, respectively. Values shown are means and standard errors. Statistically significant differences for experimental comparisons are indicated by different small letters [analysis of variance (ANOVA) and Tukey’s test, *P* < 0.05; DPS7.05].

In order to further study the *HaCAN* role on AMP expressions, gram-negative *E. coli* was injected into larvae fed with 50 μM FK506 for two days prior. A 1 h treatment with *E. coli* treatment caused significant increases in AMPs’ expressions in the fat body and midgut tissues. Interestingly, the application of FK506 significantly counteracted *E. coli*–induced AMP expression in the fat body and midgut tissues (Figure 5). While 1 h-*E. coli* treatment can significantly induced cecropin D expression in the epidermis, FK506 treatment did not significantly hinder this expression (Figure 5). Interestingly, 1 h treatments both *E. coli* and FK506 had no effect on gloverin expression in the epidermis (Figure 5). Unlike cecropin D and gloverin, the expression pattern of attacin in the epidermis was similar to that the expression pattern seen in the midgut and fat body (Figure 5).

**FIGURE 6:**
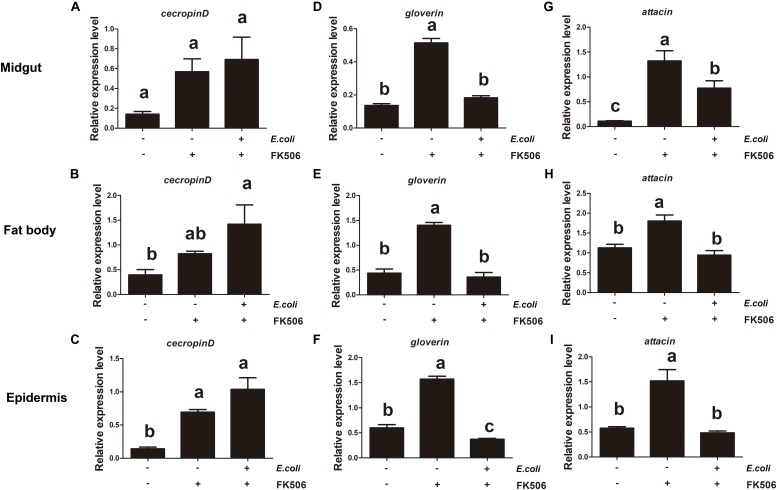
Expression levels of endogenous antimicrobial peptides (cecropin D, attacin, and gloverin) in the midgut, fat body, and epidermis in response to *Escherichia coli* infection and *E. coli* + FK506 in fourth instar *H. armigera* after 3 h treatment. **(A–C)** Represent the relative expression levels of cecropin D in the migut, fat body, and epidermis, respectively. **(D–F)** Represent the relative expression levels of attacin in the migut, fat body, and epidermis, respectively. **(G–I)** Represent the relative expression levels of gloverin in the migut, fat body, and epidermis, respectively. Values shown are means and standard errors. Statistically significant differences for experimental comparisons are indicated by different small letters [analysis of variance (ANOVA) and Tukey’s test, *P* < 0.05; DPS7.05].

Longer *E. coli* (for 3 h) treatments resulted in significant increases in the expression levels of attacin and gloverin in the midgut, fat body and epidermis. The *E. coli* induced increase of these transcripts (attacin and gloverin) was inhibited by FK506 (Figure 6). Although cecropin D expression increases with *E. coli* stimulation, it was not suppressed by FK506 in the midgut, fat body or epidermis (Figure 6).

## Discussion

Many important life processes in the cell depends on protein phosphorylation/dephosphorylation, which plays an important role in many signal transduction pathways (Patarca, 1996; Lapied et al., 2009). *HaCAN* is a critical dephosphorylase and its ubiquitous expression in different instars and tissues indicates that HaCAN participates in many biological pathways (Figure 2). The importance of CAN has been reported in other species including mammals and *Drosophila* (Dijkers and O’Farrell, 2007; Jackson et al., 2018; Kweon et al., 2018). CAN shares a high amino acid identity cross different species, which indicates that CAN may have similar functions in these different species (Chen and Zhang, 2013; Zhao et al., 2018).

FK506 is a specific inhibitor of CAN that significantly inhibit CAN dephosphatase activity (Aramburu et al., 2000). In the present study, FK506 was employed to investigate the role of *CAN* on *H. armigera* development. FK506 *H. armigera* insect development by reducing the weight of larvae, prolonging development time, and reducing pupate rates (Figure 2). These results indicate that *HaCAN* plays an important role in the development of *H. armigera* larvae. Although treatments with different FK506 concentrations for 7 days exhibited no significant differences in mortality, FK506 treatment clearly affected insect survival. The present study also revealed that *HaCAN* can regulate AMP’s expression, however, the decrease in *HaCAN*-regulated AMPs expression alone did not explain the large changes in developmental delay and pupate rate reduction. Thus, the functions of *HaCAN* needed to be further investigated.

In order to survive, insects must defend themselves against various pathogens. *Drosophila* employ two pathways (Toll and IMD) against pathogens (Kaneko and Silverman, 2005). Gram-negative bacteria infection promotes the activation of the relish transcription factor through the IMD pathway in *Drosophila* (Dijkers and O’Farrell, 2007). The expressions of the AMPs attacin, cecropin, and gloverin depend on relish expression in insects (Meng et al., 1999; Xu et al., 2012; Yang et al., 2015). Our results demonstrate that *E. coli* can induce the expression of these AMPs via the IMD pathway. However, *E. coli* induced AMP expression can be neutralized by FK506, which indicating that CAN acts as an up-stream regulator of AMP expression. AMP expression depends on the relish transcription factor and our studies confirmed that HaCAN interacts with Harelish (Figure 3). These results provide evidence for the first time that *HaCAN* can activate relish through the IMD pathway to regulate the expression of cecropin D, attacin, and gloverin under gram-negative bacteria infection. Similar results were also found in *Drosophila*, in which CAN acted on relish during infection (Li and Dijkers, 2015).

*Drosophila* utilizes two distinct pathways to express AMP genes (Kaneko and Silverman, 2005). Infection with gram-negative bacteria promotes the activation of the relish transcription factor through the IMD pathway in *Drosophila* (Dijkers and O’Farrell, 2007). The Toll pathway provides defense against gram-positive bacteria or fungal infection. Activation of the Toll pathway leads to expression of Dorsal and Dif transcription factors, which in turn up-regulate expression of AMP transcripts (Lemaitre et al., 1996, 1997). Our results show that the expression of cecropin D is regulated in part by the IMD pathway, which suggest that *HaCAN* may participate in the Toll pathway or another immune mechanism. These pathways contribute to the expression of different AMPs by activating different downstream transcription factors (Zhang et al., 2012; Zeng et al., 2019). It may be interesting to find other transcription factors that interact with *HaCAN*, and how these transcription factors function within immune response in *H. armigera*. The role of *HaCAN* in the immune response must also be further investigated in future studies.

Inhibition of *HaCAN* activity by FK506 significantly affects the development of cotton bollworms, indicating that FK506 may be used as asynergistic agent for pesticides. Moreover, plant-mediated RNAi shows great potential in crop protection. This technology relies on plants to stably express double-stranded RNAs that target essential genes in pest insects (Mamta and Rajam, 2017; Zhang et al., 2017). RNAi-mediated silencing of insect genes took part in various physiological processes were found to be detrimental to the growth, development and survival of these pest insects (Xiong et al., 2013; Zhang J. et al., 2015; Chikate et al., 2016). *HaCAN* inhibition significantly affects the development of insects (approximately 70% mortality rate with 50 μM FK506 in *H. armigera*) (Figure 1). It has also been reported that *HaCAN* can regulate sex pheromone production and change mating behavior (Zhao et al., 2018). An interesting next step will be to study RNAi of *HaCAN*, as a technology to control *H. armigera*.

## Data Availability Statement

All datasets generated for this study are included in the manuscript/supplementary files.

## Author Contributions

JW, MD, and SA conceived and designed the experiments, wrote the manuscript, and shared the microscopic observations and writing responsibilities. LL, SYao, SY, and SZ performed the experiments. JW, MD, LL, SYao, and SA analyzed the data. All authors have read and approved the manuscript for publication.

## Conflict of Interest

The authors declare that the research was conducted in the absence of any commercial or financial relationships that could be construed as a potential conflict of interest.

## References

[B1] Aramburu J. Rao A. Klee C. B. (2000). Calcineurin: from structure to function. *Curr. Top. Cell. Regul.* 36 237–295. 10.1016/s0070-2137(01)80011-x 10842755

[B2] Ballesteros-Martinez C. Mendez-Barbero N. Montalvo-Yuste A. Jensen B. M. Gomez-Cardenosa A. Klitfod L. (2017). Endothelial regulator of calcineurin 1 promotes barrier integrity and modulates histamine-induced barrier dysfunction in anaphylaxis. *Front. Immunol.* 8:1323. 10.3389/fimmu.2017.01323 29104573 PMC5655011

[B3] Bueno O. F. Brandt E. B. Rothenberg M. E. Molkentin J. D. (2002). Defective T cell development and function in calcineurin A -deficient mice. *Proc. Natl. Acad. Sci. U.S.A.* 99 9398–9403. 10.1073/pnas.152665399 12091710 PMC123152

[B4] Bustin S. A. Benes V. Garson J. A. Hellemans J. Huggett J. Kubista M. (2009). The MIQE guidelines: minimum information for publication of quantitative real-time PCR experiments. *Clin. Chem.* 55 611–622. 10.1373/clinchem.2008.112797 19246619

[B5] Chang K. T. Min K.-T. J. (2005). *Drosophila melanogaster* homolog of down syndrome critical region 1 is critical for mitochondrial function. *Nat. Neurosci.* 8 1577–1585. 10.1038/nn1564 16222229

[B6] Chang K. T. Shi Y.-J. Min K.-T. J. (2003). The *Drosophila* homolog of down’s syndrome critical region 1 gene regulates learning: implications for mental retardation. *Proc. Natl. Acad. Sci. U.S.A.* 100 15794–15799. 10.1073/pnas.2536696100 14668437 PMC307647

[B7] Chen X. E. Zhang Y. J. (2013). Molecular cloning and characterization of the calcineurin subunit A from *Plutella xylostella*. *Int. J. Mol. Sci.* 14 20692–20703. 10.3390/ijms141020692 24132154 PMC3821638

[B8] Chikate Y. R. Dawkar V. V. Barbole R. S. Tilak P. V. Gupta V. S. Giri A. P. (2016). RNAi of selected candidate genes interrupts growth and development of *Helicoverpa armigera*. *Pestic. Biochem. Physiol.* 133 44–51. 10.1016/j.pestbp.2016.03.006 27742360

[B9] Dijkers P. F. O’Farrell P. H. (2007). *Drosophila* calcineurin promotes induction of innate immune responses. *Curr. Biol.* 17 2087–2093. 10.1016/j.cub.2007.11.001 18060786 PMC2180389

[B10] Downes S. Kriticos D. Parry H. Paull C. Schellhorn N. Zalucki M. P. (2017). A perspective on management of *Helicoverpa armigera*: transgenic Bt cotton, IPM, and landscapes. *Pest Manag. Sci.* 73 485–492. 10.1002/ps.4461 27753247

[B11] Du M. Liu X. Ma N. Liu X. Wei J. Yin X. (2017). Calcineurin-mediated dephosphorylation of acetyl-coA carboxylase is required for pheromone biosynthesis activating neuropeptide (PBAN)-induced sex pheromone biosynthesis in *Helicoverpa armigera*. *Mol. cell. Proteomics* 16 2138–2152. 10.1074/mcp.RA117.000065 28978618 PMC5724177

[B12] Ejima A. Tsuda M. Takeo S. Ishii K. Matsuo T. Aigaki K. (2004). Expression level of sarah, a homolog of DSCR1, is critical for ovulation and female courtship behavior in *Drosophila melanogaster*. *Genetics* 168 2077–2087. 10.1534/genetics.104.029934 15611177 PMC1448750

[B13] Fetchko M. Stagljar I. (2004). Application of the split-ubiquitin membrane yeast two-hybrid system to investigate membrane protein interactions. *Methods* 32 349–362. 10.1016/j.ymeth.2003.10.010 15003597

[B14] Furman J. L. Norris C. M. (2014). Calcineurin and glial signaling: neuroinflammation and beyond. *J. Neuroinflammation* 11:158. 10.1186/s12974-014-0158-7 25199950 PMC4172899

[B15] Gajewski K. M. Wang J. Schulz R. A. (2006). Calcineurin function is required for myofilament formation and troponin I isoform transition in *Drosophila* indirect flight muscle. *Dev. Biol.* 289 17–29. 10.1016/j.ydbio.2005.09.039 16297904

[B16] Horner V. L. Czank A. Jang J. K. Singh N. Williams B. C. Puro J. (2006). The *Drosophila* calcipressin sarah is required for several aspects of egg activation. *Curr. Biol.* 16 1441–1446. 10.1016/j.cub.2006.06.024 16860744

[B17] Jackson S. Pallab B. Kiran K. Anupom B. Deepaneeta S. Harpreet K. (2018). A friend or foe: calcineurin across the gamut of neurological disorders. *ACS Cent. Sci.* 4 805–819. 10.1021/acscentsci.8b00230 30062109 PMC6062828

[B18] Kaneko T. Goldman W. E. Mellroth P. Steiner H. Fukase K. Kusumoto S. (2004). Monomeric and polymeric gram-negative peptidoglycan but not purified LPS stimulate the *Drosophila* IMD pathway. *Immunity* 20 637–649. 10.1016/s1074-7613(04)00104-9 15142531

[B19] Kaneko T. Silverman N. J. (2005). Bacterial recognition and signalling by the *Drosophila* IMD pathway. *Cell. Microbiol.* 7 461–469. 10.1111/j.1462-5822.2005.00504.x 15760446

[B20] Kang Y. J. Kusler B. Otsuka M. Hughes M. Suzuki N. Suzuki S. (2007). Calcineurin negatively regulates TLR-mediated activation pathways. *J. Immunol.* 179 4598–4607. 10.4049/jimmunol.179.7.4598 17878357

[B21] Kweon S. H. Lee J. Lim C. Choe J. (2018). High-amplitude circadian rhythms in *Drosophila* driven by calcineurin-mediated post-translational control of sarah. *Genetics* 209 815–828. 10.1534/genetics.118.300808 29724861 PMC6028259

[B22] Lapied B. Pennetier C. Apaire-Marchais V. Licznar P. Corbel V. (2009). Innovative applications for insect viruses: towards insecticide sensitization. *Trends Biotechnol.* 27 190–198. 10.1016/j.tibtech.2008.12.005 19251330

[B23] Lemaitre B. Nicolas E. Michaut L. Reichhart J. M. Hoffmann J. A. (1996). The dorsoventral regulatory gene cassette spätzle/Toll/cactus controls the potent antifungal response in *Drosophila* adults. *Cell* 86 14614–14619. 10.1016/s0092-8674(00)80172-5 8808632

[B24] Lemaitre B. Reichhart J.-M. Hoffmann J. A. (1997). *Drosophila* host defense: differential induction of antimicrobial peptide genes after infection by various classes of microorganisms. *Proc. Natl. Acad. Sci. U.S.A.* 94 14614–14619. 10.1073/pnas.94.26.14614 9405661 PMC25070

[B25] Li Y. X. Dijkers P. F. (2015). Specific calcineurin isoforms are involved in *Drosophila* Toll immune signaling. *J. Immunol.* 194 168–176. 10.4049/jimmunol.1401080 25429067

[B26] Liu J. Albers M. W. Wandless T. J. Luan S. Alberg D. G. Belshaw P. J. (1992). Inhibition of T cell signaling by immunophilin-ligand complexes correlates with loss of calcineurin phosphatase activity. *Biochemistry* 31 3896–3901. 10.1021/bi00131a002 1373650

[B27] Liu J. Farmer J. D. Lane W. S. Friedman J. Schreiber S. L. (1991). Calcineurin is a common target of Cyclophilin-Cyclosporin A and FKBP-FK506 complexes. *Cell* 66 807–815. 10.1016/0092-8674(91)90124-h 1715244

[B28] Livak K. J. Schmittgen T. D. (2001). Analysis of relative gene expression data using real-time quantitative PCR and the 2(-Delta Delta C(T)) Method. *Methods* 25 402–408. 10.1006/meth.2001.1262 11846609

[B29] Mamta B. Rajam M. V. (2017). RNAi technology: a new platform for crop pest control. *Physiol. Mol. Biol. Plants* 23 487–501. 10.1007/s12298-017-0443-x 28878489 PMC5567704

[B30] Meng X. Khanuja B. S. Ip Y. T. (1999). Toll receptor-mediated *Drosophila* immune response requires Dif, an NF- κB factor. *Genes Dev.* 13 792–797. 10.1101/gad.13.7.792 10197979 PMC316597

[B31] Patarca R. J. (1996). Protein phosphorylation and dephosphorylation in physiologic and oncologic processes. *Crit. Rev. Oncog.* 7 343–432. 10.1615/critrevoncog.v7.i5-6.20 9467665

[B32] Sakai T. Aigaki T. J. (2010). The *Drosophila* calcineurin regulator, Sarah, is involved in male courtship. *Neuroreport* 21 985–988. 10.1097/WNR.0b013e32833eaade 20736865

[B33] Schmittgen T. D. Livak K. J. (2008). Analyzing real-time PCR data by th comparative CT method. *Nat. Protoc.* 3 1101–1108. 10.1038/nprot.2008.73 18546601

[B34] Shaw J. L. Chang K. T. Lu B. (2013). Nebula/DSCR1 upregulation delays neurodegeneration and protects against APP-Induced axonal transport defects by restoring calcineurin and GSK-3β signaling. *PLoS Genet.* 9:e1003792. 10.1371/journal.pgen.1003792 24086147 PMC3784514

[B35] Takeo S. Hawley R. S. Aigaki T. (2010). Calcineurin and its regulation by Sra/RCAN is required for completion of meiosis in *Drosophila*. *Dev. Biol.* 344 957–967. 10.1016/j.ydbio.2010.06.011 20561515

[B36] Wei J. Zhang L. Yang S. Xie B. An S. Liang G. (2018). Assessment of the lethal and sublethal effects by spinetoram on cotton bollworm. *PLoS One* 13:e0204154. 10.1371/journal.pone.0204154 30216388 PMC6138415

[B37] Xiong Y. Zeng H. Zhang Y. Xu D. Qiu D. (2013). Silencing the HaHR3 gene by transgenic plant-mediated RNAi to disrupt *Helicoverpa armigera* development. *Int. J. Biol. Sci.* 9 370–381. 10.7150/ijbs.5929 23630449 PMC3638292

[B38] Xu X. Zhong X. Yi H. Yu X. (2012). *Manduca sexta* gloverin binds microbial components and is active against Bacteria and Fungi. *Dev. Comp. Immunol.* 38 275–284. 10.1016/j.dci.2012.06.012 22858411 PMC3443299

[B39] Yang J. Wang X. Tang S. Shen Z. Wu J. (2015). Peptidoglycan recognition protein S2 from silkworm integument: characterization, microbe-Induced expression, and involvement in the immune-deficiency pathway. *J. Insect Sci.* 15:20. 10.1093/jisesa/iev007 25797797 PMC4535147

[B40] Yoshiga T. Yokoyama N. Imai N. Ohnishi A. Moto K. Matsumoto S. (2002). cDNA cloning of calcineurin heterosubunits from the pheromone gland of the silkmoth, *Bombyx mori*. *Insect Biochem. Mol. Biol.* 32 477–486. 10.1016/s0965-1748(01)00125-4 11886782

[B41] Yu Y. Li Y. Zhang Y. (2015). Screening of APP interaction proteins by DUALmembrane yeast two-hybrid system. *Int. J. Exp. Pathol.* 8 2802–2808. 26045787 PMC4440096

[B42] Zeng L. Li Z. Liu L. (2019). Research progress in the immunity of insects and the immune mechanisms of five important invasive insects. *J. Plant Prot.* 46 6–16.

[B43] Zhang J. Khan S. A. Hasse C. Ruf S. Heckel D. G. Bock R. (2015). Pest control. Full crop protection from an insect pest by expression of long double-stranded RNAs in plastids. *Science* 347 991–994. 10.1126/science.1261680 25722411

[B44] Zhang S. An S. Li Z. Wu F. Yang Q. Liu Y. (2015). Identification and validation of reference genes for normalization of gene expression analysis using qRT-PCR in *Helicoverpa armigera* (Lepidoptera: Noctuidae). *Gene* 555 393–402. 10.1016/j.gene.2014.11.038 25447918

[B45] Zhang J. Khan S. A. Heckel D. G. (2017). Next-generation insect-resistant plants RNAi-mediated crop protection. *Trends Biotechnol.* 8 871–882. 10.1016/j.tibtech.2017.04.009 28822479

[B46] Zhang M. Chu Y. Zhao Z. An C. (2012). Progress in the molecular mechanisms of the innate immune responses in insects. *Acta Entomologica Sinica* 55 1221–1229.

[B47] Zhao W. Li L. Zhang Y. Liu X. Wei J. Xie Y. (2018). Calcineurin is required for male sex pheromone biosynthesis and female acceptance. *Insect Mol. Biol.* 27 373–382. 10.1111/imb.12379 29465818

